# Preoperative Neutrophil-to-Lymphocyte Ratio as a Predictive and Prognostic Factor for High-Grade Serous Ovarian Cancer

**DOI:** 10.1371/journal.pone.0156101

**Published:** 2016-05-20

**Authors:** Zheng Feng, Hao Wen, Rui Bi, Xingzhu Ju, Xiaojun Chen, Wentao Yang, Xiaohua Wu

**Affiliations:** 1 Department of Gynecological Oncology, Fudan University Shanghai Cancer Center, Shanghai, 200032, China; 2 Department of Oncology, Shanghai Medical College, Fudan University, Shanghai, 200032, China; 3 Department of Pathology, Fudan University Shanghai Cancer Center, Shanghai, 200032, China; University of Nebraska Medical Center, UNITED STATES

## Abstract

**Objective:**

We aimed to demonstrate the clinical and prognostic significance of the preoperative neutrophil-to-lymphocyte ratio (NLR) in high-grade serous ovarian cancer (HGSC).

**Methods:**

We retrospectively investigated 875 patients who underwent primary staging or debulking surgery for HGSC between April 2005 and June 2013 at our institution. None of these patients received neoadjuvant chemotherapy. NLR was defined as the absolute neutrophil count divided by the absolute lymphocyte count. Progression-free survival (PFS) and overall survival (OS) were analyzed with the Kaplan-Meier method and log-rank tests for univariate analyses. For multivariate analyses, Cox regression analysis was used to evaluate the effects of the prognostic factors, which were expressed as hazard ratios (HRs).

**Results:**

The NLRs ranged from 0.30 to 24.0. The median value was 3.24 and used as the cutoff value to discriminate between the high-NLR (≥3.24) and low-NLR (<3.24) groups. A high preoperative NLR level was associated with an advanced FIGO stage, increased CA125 level, more extensive ascites, worse cytoreduction outcome and chemoresistance. For univariate analyses, a high NLR was associated with reduced PFS (p<0.001) and OS (p<0.001). In multivariate analyses, a high NLR was still an independent predictor of PFS (p = 0.011), but not OS (p = 0.148).

**Conclusion:**

Our study demonstrated that NLR could reflect tumor burden and clinical outcomes to a certain extent and should be regarded as a predictive and prognostic parameter for HGSC.

## Introduction

Ovarian cancer is one of the most commonly diagnosed and lethal diseases among females, and there were 238,700 estimated new cases and 151,900 deaths in 2012 around the world[1]. After primary treatment including staging or debulking surgery and platinum-based adjuvant chemotherapy, half of the patients will relapse within 16 months, and the 5-year overall survival rate is under 50%[2]. Therefore, effective biomarkers for individualized prediction of treatment outcomes and prognosis are urgently required.

Inflammation plays an important role during cancer initiation and progression, and the prognostic value of systemic inflammatory response (SIR) markers has been of paramount interest[3]. The neutrophil-to-lymphocyte ratio (NLR), one of the most common SIR markers, has been used to predict clinical outcomes and prognoses in various cancers[4–7]. Limited data have also shown the application of NLR in ovarian cancer[8–10]. However, ovarian cancer is not a single disease, it is a group of heterogeneous tumors based on distinctive morphologic and molecular genetic features[11]. Previous studies have combined all disease subtypes within small sample sizes, which failed to individually evaluate the clinical and prognostic values of NLR according to the histologic types.

Because the vast majority of ovarian cancers are high-grade serous ovarian cancer (HGSC), the purpose of our study was to investigate the clinical and prognostic significance of preoperative NLR in a large mono-institutional study of Chinese patients with HGSC.

## Methods

### Clinical Data

This study was conducted according to the Declaration of Helsinki and was approved by the Committee at Fudan University Shanghai Cancer Center. Written informed consent was obtained from all individual participants included in the study.

We retrospectively investigated 875 patients who underwent primary staging or debulking surgery for HGSC between April 2005 and June 2013 at Fudan University Shanghai Cancer Center. Patients were excluded if they had received neoadjuvant therapy, had been treated for recurrent disease, or were found to have other histological diagnoses after a pathological review. The histological diagnoses were based on the WHO criteria, and all microscopic slides were reviewed by two experienced gynecologic pathologists.

Clinical and pathological data were obtained from medical records, cancer registries, and pathology reports. Patient characteristics including age, FIGO stage (version 2000), preoperative laboratory data (CA125 level, preoperative neutrophil count, lymphocyte count), presence of ascites, surgical outcomes (R0, R1 *vs* R2), date of surgery, date of progression or recurrence, date of last follow-up, and patient’s disease status at last contact, were collected. All patients were followed up with until December 31^st^, 2014.

NLR was defined as the absolute neutrophil count divided by the absolute lymphocyte count. Preoperative blood samples from the patient were drawn by antecubital venipuncture within 1 week prior to the operation.

R0 was defined as the absence of macroscopic residual disease (RD) after surgery. R1 was defined as a maximal diameter of the macroscopic residual disease after a cytoreduction of <1 cm. R2 was defined as a maximal diameter of residual disease of ≥1 cm. Chemosensitivity was defined as a time interval of 6 months or longer between the completion of platinum-based chemotherapy and the detection of relapse. Chemoresistance was defined as disease progression during adjuvant chemotherapy or within the 6-month interval between the completion of chemotherapy and detection of a relapse.

PFS was defined as the time interval from the date of primary surgery to the date of disease progression or recurrence. OS was defined as the time interval from the date of the primary surgery to the date of death or the last follow-up.

### Statistical Analyses

SPSS software (version 21.0) and GraphPad Prism (version 6.0) were used for statistical analyses. Descriptive statistics were used for the demographic data and were summarized as the medians with the interquartile ranges (IQRs) or ranges, or the frequencies with the percentages. The categorical data were compared with chi-square or Fisher’s exact tests as appropriate. The PFS and OS were analyzed with the Kaplan-Meier method, and log-rank tests were used in the univariate analyses. For the multivariate analyses, Cox regression analysis was used to evaluate the effects of the prognostic factors, which were expressed as hazard ratios (HRs). P<0.05 was considered statistically significant, and all reported P values were 2-sided.

## Results

### Patient Characteristics and Their Correlations with NLR

The patient characteristics are shown in Table 1. The majority of the patients (800/875) had advanced stage (III-IV). The median (interquartile range, IQR) neutrophil count was 4.6 (3.6–5.7)×10^9^/L, and the median (IQR) lymphocyte count was 1.4 (1.1–1.8)×10^9^/L. NLR ranged from 0.30 to 24.0, with a median level of 3.24.

**Table 1:** Patient Characteristics (n = 875).

Age at diagnosis, median (range), years	56 (30–90)	
Follow-up time, median (range), months	29 (1–115)	
**Vital status**		
Died	345	39.4%
Alive	448	51.2%
Censored	82	9.4%
**Family history**		
Yes	230	26.3%
No	643	73.7%
**CA125 level**		
<500 U/ml	193	22.6%
≥500 U/ml	662	77.4%
**Ascites**		
No	99	11.3%
<500ml	146	16.7%
≥500ml	629	72.0%
**FIGO Stage**		
Early (I, II)	75	8.6%
Advanced (III, IV)	800	91.4%
**Cytoreduction**		
R0	272	31.1%
R1	434	49.6%
R2	169	19.3%
**Chemosensitivity**		
Yes	568	66.9%
No	237	27.9%
NA	44	5.2%
**N (Median, IQR) (×10**^**9**^**/L)**	4.6(3.6–5.7)	
**L (Median, IQR) (×10**^**9**^**/L)**	1.4(1.1–1.8)	
**Neutrophil-to-lymphocyte Ratio, NLR**	3.24(2.31–4.50)	

Correlations of the clinical characteristics with preoperative NLR are summarized in Table 2. The median value 3.24 was used as the cutoff value to discriminate between the high-NLR (≥3.24) and low-NLR (<3.24) groups. A high preoperative NLR level was associated with an advanced FIGO stage (p<0.001), an increased CA125 level (p<0.001) and more extensive ascites (p<0.001).

**Table 2:** Patient clinical parameters and NLR.

Parameters	Low NLR	High NLR	P-value
**Age**			
<56(427)	193(45.2%)	234(54.8%)	0.010
≥56(448)	242(54.0%)	206(46.0%)	
**FIGO Stage**			
Early(75)	55(73.3%)	20(26.7%)	<0.001
Advanced(800)	380(47.5%)	420(52.5%)	
**CA125 level**			
<500U/ml(193)	140(72.5%)	53(27.5%)	<0.001
≥500U/ml(662)	287(43.4%)	375(56.6%)	
**Ascites**			
No(99)	77(77.8%)	22(22.2%)	<0.001
<500ml(146)	99(67.8%)	47(32.2%)	
≥500ml(629)	258(41.0%)	371(59.0%)	
**Cytoreduction**			
R0(272)	173(63.6%)	99(36.4%)	<0.001
R1(434)	194(44.7%)	240(55.3%)	
R2(169)	68(40.2%)	101(59.8%)	
**Chemosensitivity**			
Yes(568)	311(54.8%)	257(45.2%)	<0.001
No(237)	94(39.7%)	143(60.3%)	

### Treatment Outcomes, Survival Analysis, and Their Correlations with NLR

After primary surgery, 272 (31.1%) of the patients were debulked to R0 and 434 (49.6%) were debulked to ≤1 cm with macroscopic disease (R1). As the extent of residual disease increased, the number of patients who presented with high NLR increased (p<0.001).

In our study, 849 (97.0%) patients had received platinum-based adjuvant chemotherapy after primary surgery. Among these patients, 568 (66.9%) were chemosensitive (Table 1). A greater proportion of the patients with chemoresistant disease had a high NLR (60.3% *vs* 45.2%, p<0.001) compared with the chemosensitive patients.

The median follow-up time was 29 (1–115) months. 104 (11.9%) women experienced disease progression during adjuvant chemotherapy, 499 (57.0%) patients exhibited documented recurrence, and 345 (39.4%) deaths were documented. The median (95% CI) PFS was 18 (16.8–19.2) months, and the median (95% CI) OS was 58 (51.4–64.6) months, respectively.

The known negative influences of an advanced FIGO stage (p<0.001 and <0.001, respectively), the presence of residual disease (R0 *vs* R1+R2: p<0.001 and <0.001, respectively), and chemoresistance (p<0.001 and <0.001, respectively) for PFS and OS were confirmed by univariate analyses.

**Fig 1:**
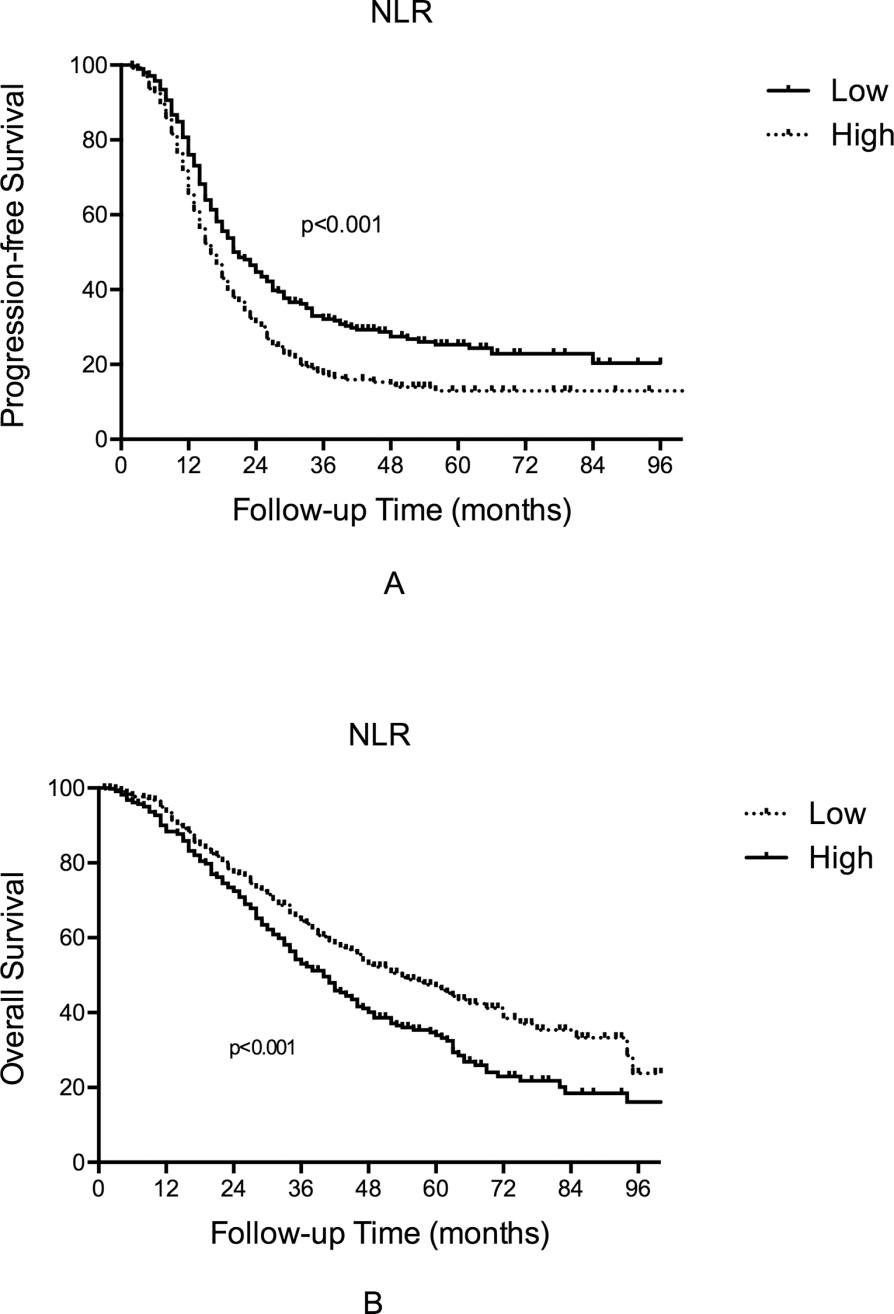
Kaplan-Meier curve of PFS and OS stratified by NLR level.

In the univariate analysis, a high NLR was associated with impaired PFS (16.0 (14.4–17.6) *vs* 21.0 (18.2–23.8) months, p<0.001, Fig 1A). In the multivariate analysis with adjustments for age, FIGO stage, cytoreduction outcome and chemosensitivity status, high NLR was also an independent predictor of a poorer PFS (HR = 1.250, 95% CI, 1.052–1.484, p = 0.011; Table 3).

**Table 3:** Multivariable analysis of factors associated with PFS.

Characteristics	HR	95% CI	P value
**Age as continuous variable**	1.002	0.994–1.011	0.601
**FIGO Stage**			
Early	Referent		
Advanced	2.353	1.543–3.587	<0.001
**Cytoreduction**			
R0	Referent		
R1	1.240	0.999–1.540	0.051
R2	1.343	1.027–1.756	0.031
**Chemosensitivity**			
No	Referent		
Yes	0.082	0.065–0.103	<0.001
**NLR**			
Low	Referent		
High	1.250	1.052–1.484	0.011

A high NLR was also associated with a shorter OS in the univariate analysis (45.0 (38.9–51.1) *vs* 69.0 (59.1–78.9) months, p<0.001, Fig 1B). In the multivariate analysis with adjustments for age, FIGO stage, cytoreduction outcome and chemosensitivity status, NLR was not independently associated with OS (HR = 1.189, 0.940–1.504, p = 0.148; Table 4).

**Table 4:** Multivariable analysis of factors associated with OS.

Characteristics	HR	95% CI	P value
**Age as continuous variable**	1.012	1.000–1.025	0.056
**FIGO Stage**			
Early	Referent		
Advanced	3.333	1.593–6.975	0.001
**Cytoreduction**			
R0	Referent		
R1	1.718	1.241–2.378	0.001
R2	1.294	0.880–1.904	0.190
**Chemosensitivity**			
No	Referent		
Yes	0.197	0.155–0.250	<0.001
**NLR**			
Low	Referent		
High	1.189	0.940–1.504	0.148

## Discussion

In this large mono-institutional study, we demonstrated that the preoperative NLR was associated with the clinical characteristics and treatment outcomes. Additionally, a high NLR was correlated with impaired PFS and OS in Chinese patients with HGSC.

Links between inflammation and cancer development have garnered much interest in the past few decades[3]. It's well established that hematological markers of systemic inflammation (including C-reactive protein, albumin, neutrophils and so on) could help predict survival in patients with various types of cancer[12–15]. Among these predictors, NLR is a reproducible and widely available laboratory hematological marker in our routine clinical practice. Several studies have shown that an elevated NLR was associated with impaired prognosis in patients with many cancers[4–7]. However, the results from other studies were inconsistent[16–18]. For instance, Suppan et al[18] found no predictive or prognostic value in breast cancer patients.

The data on ovarian cancer are quite limited. Cho et al[9] studied the NLR levels in epithelial ovarian cancer patients compared with those in patients with benign ovarian tumors, benign gynecologic disease, and healthy controls. They found that NLR levels could help identify ovarian cancer cases (sensitivity = 66.1% and specificity = 82.7%) and predict OS (HR = 8.42 (1.09–64.84), p = 0.041). Williams et al[19] have further confirmed that higher NLR levels were associated with various clinical characteristics (including higher tumor stage and grade, the presence of ascites and so on) and impaired prognosis in 519 ovarian cancer patients. Furthermore, Raungkaewmanee et al[10] found no correlation between NLR and PFS or OS in ovarian cancer patients.

Our findings are in accordance with several previously published data. We have proven that high NLR levels were associated with an advanced FIGO stage, an increased CA125 level and more extensive ascites. Moreover, our study demonstrated that lower NLR level was correlated with better cytoreduction outcome. Therefore, NLR could reflect tumor burden and be used to predict debulking outcomes for ovarian cancer patients.

In addition, we found that greater proportions of the HGSC patients with chemoresistant disease had higher NLR levels compared with the chemosensitive patients. Wang et al[17] have drawn similar conclusion that patients with higher NLR level had significantly lower complete chemotherapy response rates. These above results indicate the potential role of NLR to predict ovarian cancer patient chemosenstivity.

Furthermore, for prognostic analysis, the NLR level was a negative independent predictor of PFS (p = 0.011), but not OS (p = 0.148) as a dichotomous variable. However, NLR was a negative independent predictor of both PFS and OS as a continuous variable. This may be due to the selection of cutoff value to discriminate between the high-NLR and low-NLR groups. In previous studies, the cutoff value was determined by several methods (such as the interquartile level, receiver operating curve (ROC) for cytoreduction outcomes, ROC for OS and so on)[9, 10, 15, 20]. Thus, it's difficult to evaluate which method is the best. Our study selected the median NLR level as the cutoff value, and this was convenient to dichotomize NLR into low and high groups with equal cases. Prevalence studies of the NLR levels in healthy women should be completed to determine the normal range of NLR, and this would help to define high NLR levels.

A limitation in our study is that it was a retrospective study that depended on accurate documentation, and thus, the potential for recall bias existed. Notwithstanding its limitation, this was a mono-institutional retrospective study that involved a group of homogenous patients with the same histology who underwent similar treatment strategies. Our study demonstrated that the NLR could reflect tumor burden and clinical outcomes to a certain extent, and should be regarded as a predictive and prognostic parameter for HGSC.
